# Effects of Liposomal Formulation of Citicoline in Experimental Diabetes-Induced Retinal Neurodegeneration

**DOI:** 10.3390/ijms19082458

**Published:** 2018-08-20

**Authors:** Patricia Bogdanov, Joel Sampedro, Cristina Solà-Adell, Olga Simó-Servat, Carla Russo, Luisa Varela-Sende, Rafael Simó, Cristina Hernández

**Affiliations:** 1 Institut de Recerca Hospital UniversitariValld’Hebron (VHIR), 08035 Barcelona, Spain; patricia.bogdanov@vhir.org (P.B.); joel.sampedro@vhir.org (J.S.); cristina.sola@vhir.org (C.S.-A.); olga.simo@vhir.org (O.S.-S.); rafael.simo@vhir.org (R.S.); 2 CIBERDEM (Instituto de Salud Carlos III), 28029 Madrid, Spain; 3 Department of Medicine, Universitat Autònoma de Barcelona, 08193 Barcelona, Spain; 4 R&D Department, Omikron Italia Srl, 00197 Roma, Italy; c.russo@omikronitalia.it; 5 Clinical Research Department, OPKO Health Spain S.L., 08908 Barcelona, Spain; LVarela@opko.com

**Keywords:** citicoline, diabetic retinopathy, retinal neurodegeneration, db/db mouse

## Abstract

Diabetic retinopathy (DR) has been classically considered a microcirculatory disease of the retina. However, there is growing evidence to suggest that retinal neurodegeneration is also an early event in the pathogenesis of DR. Citicoline has been successfully used as a neuroprotective agent in the treatment of glaucoma but their effects on DR remain to be elucidated. On this basis, the main aim of the present study was to evaluate the effect of topical administration of citicoline in liposomal formulation on retinal neurodegeneration in db/db mouse and to investigate the underlying mechanisms of action. The treatment (citicoline or vehicle) was topically administered twice daily for 15 days. Retinal analyses were performed in vivo by electroretinography and ex vivo by using Western blot and immunofluorescence measurements. We found that the liposomal formulation of citicoline prevented glial activation and neural apoptosis in the diabetic retina. The main mechanism implicated in these beneficial effects were the inhibition of the downregulation of synaptophysin and its anti-inflammatory properties by means of preventing the upregulation of NF-κB and TNF-α (Tumor Necrosis Factor α) induced by diabetes. Overall, these results suggest that topical administration of citicoline in liposomal formulation could be considered as a new strategy for treating the early stages of DR.

## 1. Introduction

Diabetic retinopathy (DR) has been classically considered a microcirculatory disease of the retina. However, there is growing evidence to suggest that retinal neurodegeneration is an early event in the pathogenesis of DR which participates in the microcirculatory abnormalities that occur in DR [1,2,3,4]. In fact, the American Diabetes Association has recently defined DR as a highly specific neurovascular complication [5]. Thus, the use of neuroprotective agents opens up a new field of research based on neuroprotection for preventing or arresting the development of DR [6]. However, in these early stages of DR, intravitreous injections are inappropriately invasive and the use of eye drops seems a more suitable route of administration. 

Citicoline (cytidine 5′-dihosphocoline) is an endogenous compound that acts in the biosynthetic pathway of phospholipids of cell membranes, particularly phosphatidylcholine levels, and it is able to increase neurotransmitters levels in the central nervous system [7,8]. Several experimental studies have suggested that citicoline is a neuroprotective agent. Citicoline has been shown to be effective in reducing infarct volume, brain edema, and in improving neurologic deficits in stroke [9,10,11,12,13]. Citicoline also showed efficacy in Alzheimer’s disease possibly interfering on the deposition of neurotoxic proteins such as β-amyloid, and improving mental performance and brain electrical activity [14]. Furthermore, clinical studies suggest that administration of citicoline may slow down cognitive decline [15,16,17]. Recently, citicoline oral solution obtained a registration as Food for Special Medical purposes with the therapeutical indication for Glaucomatous patient pharmacologically controlled but with progressive loss of visual field.

Citicoline, by either injectable, oral or eye drop administration, has been used as a neuroprotective agent in the treatment of glaucoma [18,19,20,21,22]. However, the effect on DR has been barely analyzed and the underlying mechanism remains to be elucidated [23]. In addition, a new preservative-free liposomal eye drop formulation has been developed. This new formulation apart from avoiding the secondary effect of preservatives (i.e., benzalkonium chloride), maintains the capacity of citicoline to reach the retinal ganglion cells [24].

On this basis, the main aim of the present study was to evaluate the effect of topical administration on this new liposomal formulation of citicoline on retinal neurodegeneration in db/db mice, an experimental model which reproduces very well the early stages of DR. In addition, the potential mechanisms involved in its eventual beneficial neuroprotective action were also examined.

## 2. Results

**Figure 1:**
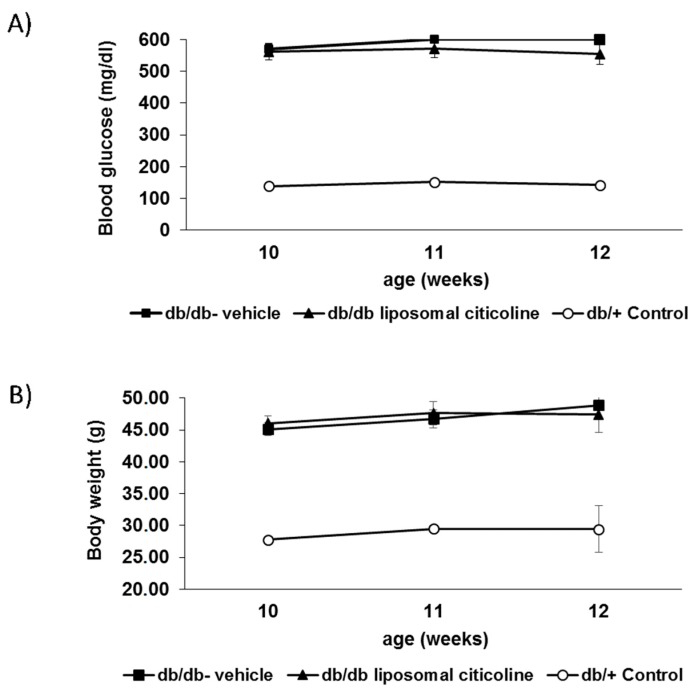
Evolution of blood glucose (**A**) and body weight (**B**) in the experimental groups.

We did not find any difference in blood glucose concentrations and body weight during the study between db/db mice treated with topical administration of citicoline and db/db mice treated with vehicle (Figure 1).

### 2.1. Topical Administration of Liposomal Formulation of Citicoline Prevents Retinal Neurodegeneration in Diabetic Mice

#### 2.1.1. Glial Activation

**Figure 2:**
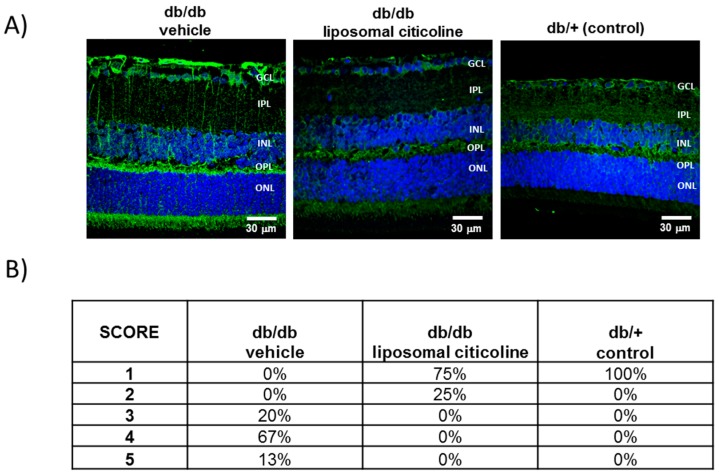
Effect of citicoline on glial activation. (**A**) Comparison of GFAP immunoreactivity (green) in the retina among representative samples from a diabetic mouse treated with vehicle, a diabetic mouse treated with citicoline, and a non-diabetic mouse. Nuclei were labeled with Hoechst (blue). ONL: outer nuclear layer; OPL: outer plexifom layer; INL: inner nuclear layer; IPL: inner plexiform layer; GCL: ganglion cell layer. Scale bars, 30 µm. (**B**) Quantification of glial activation based on the extent of GFAP staining. *n* = seven mice per group.

GFAP (Glial fibrillar acidic protein) expression was mainly confined to the retinal ganglion cell layer (GCL) in non-diabetic control mice (db/+) (Figure 2). By contrast, age-matched diabetic mice (db/db) treated with vehicle presented significantly higher GFAP expression. It must be noted that 100% of diabetic mice presented a GFAP score of ≥3. The administration of citicoline resulted in a significant decrease of reactive gliosis, being a GFAP score of ≤3 in all cases (Figure 2).

#### 2.1.2. Apoptosis

**Figure 3:**
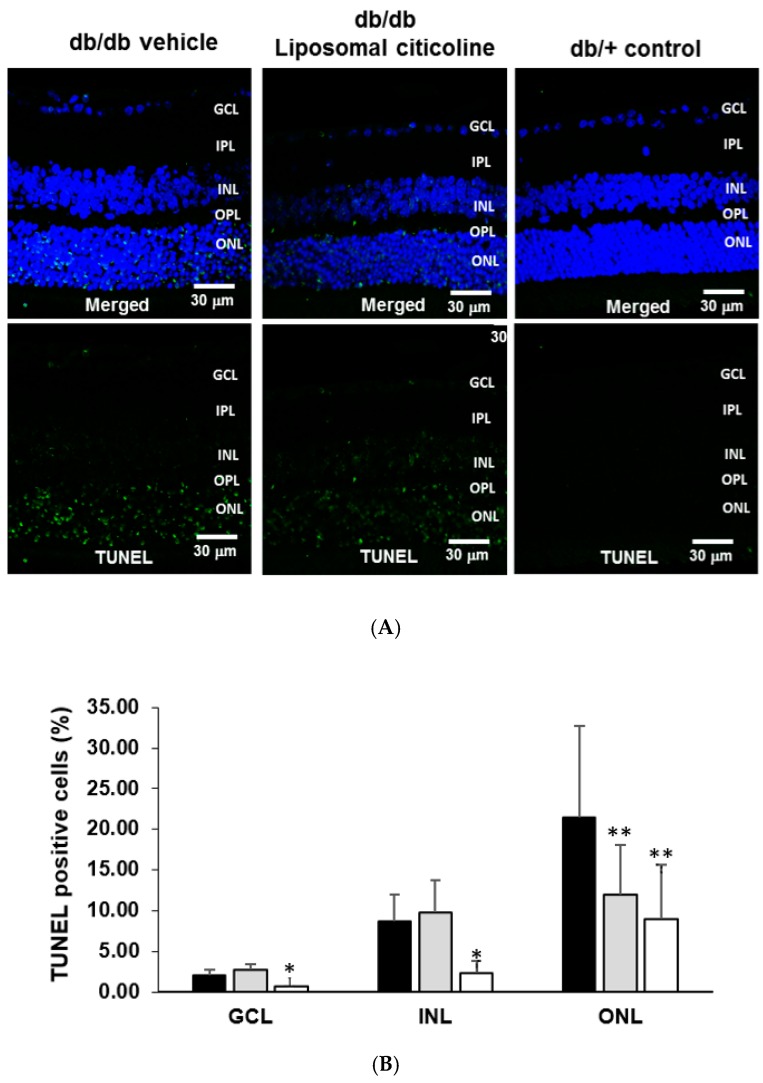
Effect of citicoline on apoptosis. (**A**) TUNEL (Terminal Transferase dUTP Nick-End Labeling) positive immunofluorescence (green) in a representative mouse from each group. Nuclei were labeled with Hoechst (blue). Scale bars, 30 µm; (**B**) percentage of TUNEL positive cells in the neuroretina. ONL: outer nuclear layer; INL: inner nuclear layer; GCL: ganglion cell layer. Black columns: db/db-vehicle; gray columns: db/db-citicoline; white columns: db/+. Results are mean ± SD. *: *p* < 0.05 in comparison with the other groups. **: *p* < 0.05 in comparison with db/db-vehicle. *n* = seven mice per group.

**Figure 4:**
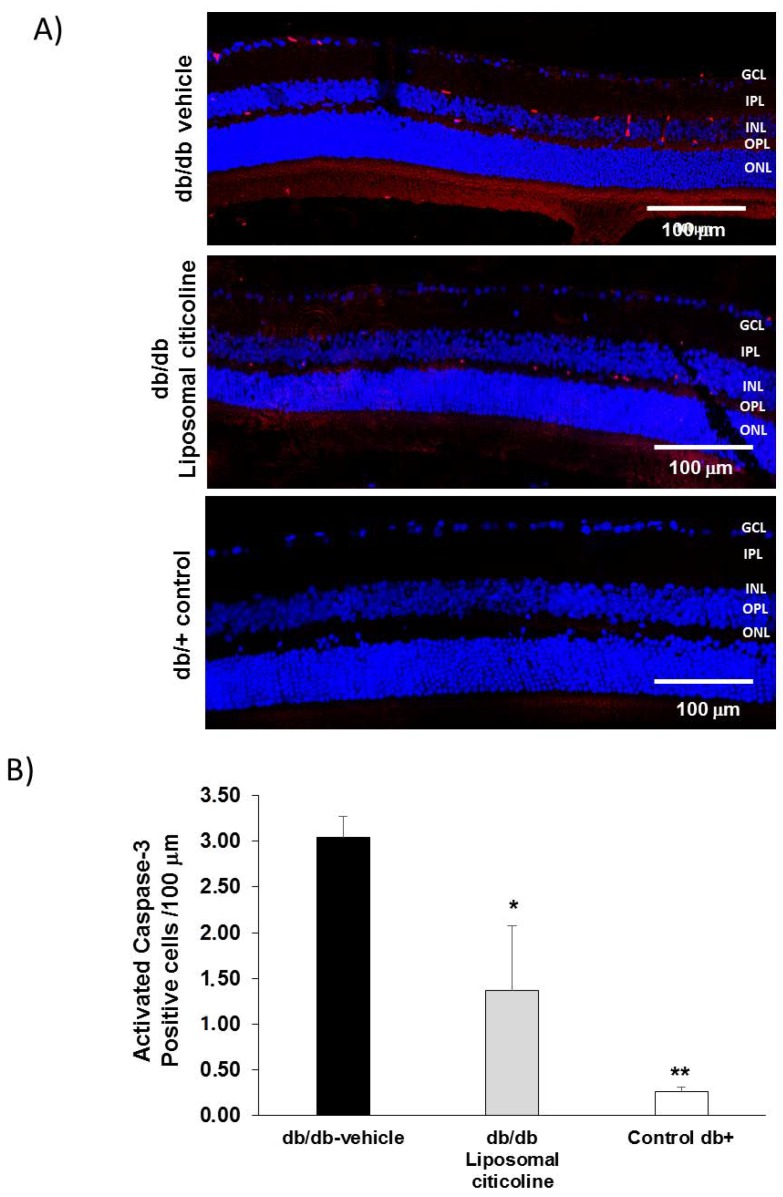
Effect of citicoline on caspase 3. (**A**) Activated Caspase 3 immunofluorescence (red) in a representative mouse from each group. Scale bars, 100 µm. Nuclei were labeled with Hoechst (blue). ONL: outer nuclear layer; OPL: outer plexiform layer; INL: inner nuclear layer; IPL: inner plexiform layer; GCL: ganglion cell layer; (**B**) quantification of activated caspase 3 immunofluorescence. Results are mean ± SD. *: *p* < 0.05 in comparison with the other groups. **: *p* < 0.05 in comparison with the other groups. *n* = seven mice per group.

The percentage of apoptotic cells in retinal layers (ONL, INL, and GCL) was significantly higher in diabetic mice in comparison to age-matched non-diabetic controls (Figure 3). Diabetic mice treated with liposomal citicoline presented a lower rate of apoptosis than diabetic mice treated with vehicle in the ONL (Figure 3). In addition, activated caspase 3 was significantly lower in db/db mice treated with citicoline in comparison with db/db mice treated with vehicle (Figure 4). Spectral domain OCT (optical coherence tomography) showed that citicoline reduces the neuroretinal thinning induced by diabetes, but we did not find any statistical difference among groups. 

#### 2.1.3. Electroretinography Abnormalities

**Figure 5:**
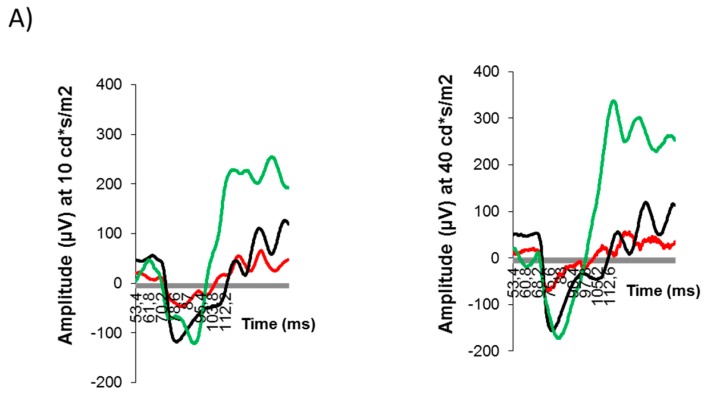

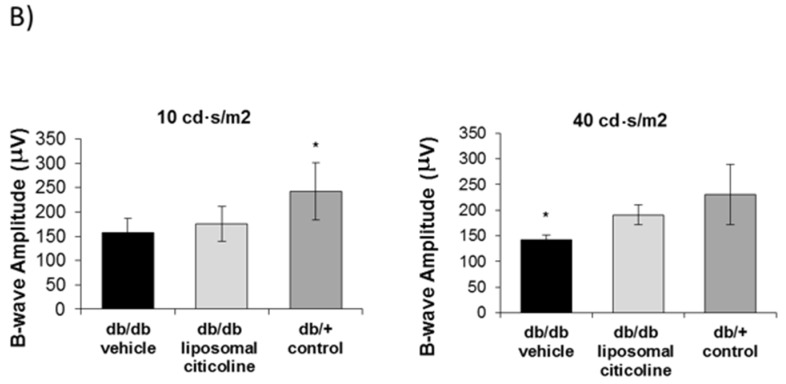
Effect of citicoline on electroretinography (ERG)abnormalities. (**A**) ERG traces in response to low (10 cd·s/m^2^) and medium (40 cd·s/m^2^) stimulus intensities in a representative mouse of each investigational group. Red line: db/db-vehicle. Black line: db/db-citicoline. Green line: db/+ (non-diabetic control); (**B**) quantitative analyses of amplitude of b-wave in db/db treated with vehicle, db/db treated with citicoline, and non-diabetic mice. Results are mean ± SD. * *p* < 0.05 in comparison with the other groups.

The average amplitude of b-wave as a function of flash intensity is displayed in Figure 5. The b-wave was significantly lower in diabetic mice at several flash intensities tested in comparison with non-diabetic mice. Treatment with citicoline was able to ameliorate these functional abnormalities induced by diabetes (Figure 5). 

### 2.2. Mechanisms Involved in Neuroprotection

#### 2.2.1. Citicoline Prevents the Downregulation of Synaptophysin Induced by Diabetes

**Figure 6:**
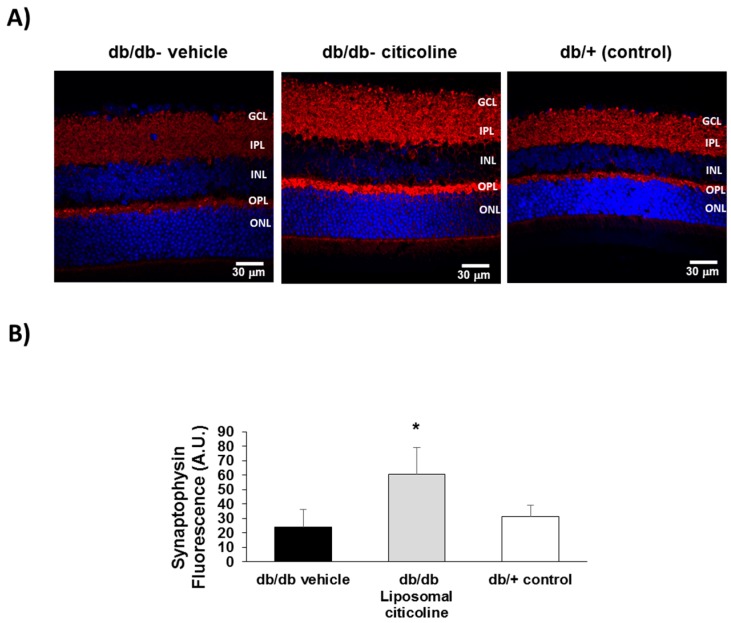
Effect of citicoline on synaptophysin. (**A**) Comparison of synaptophysin immunofluorescence (red) between representative samples from a db/db mouse treated with vehicle, a db/db mouse treated with citicoline, and a non-diabetic mouse. Nuclei were labeled with Hoechst (blue). ONL: outer nuclear layer; OPL: outer plexiform layer; INL: inner nuclear layer; IPL: inner plexiform layer; GCL: ganglion cell layer; (**B**) Quantification of synaptophysin immunofluorescence in arbitrary units (AU). Results are mean ± SD. * *p* < 0.01 vs. the other groups.

We found a downregulation in synaptophysin in retinas from diabetic mice treated with vehicle in comparison with non-diabetic mice, thus revealing a synapse loss induced by diabetes (Figure 6). Notably, topical treatment with liposomal formulation of citicoline was able not only to prevent the decrease of synaptophysin caused by diabetes but even to increase its expression. 

#### 2.2.2. Citicoline Prevents the Upregulation of NF-κB and TNF-α Induced by Diabetes

**Figure 7:**
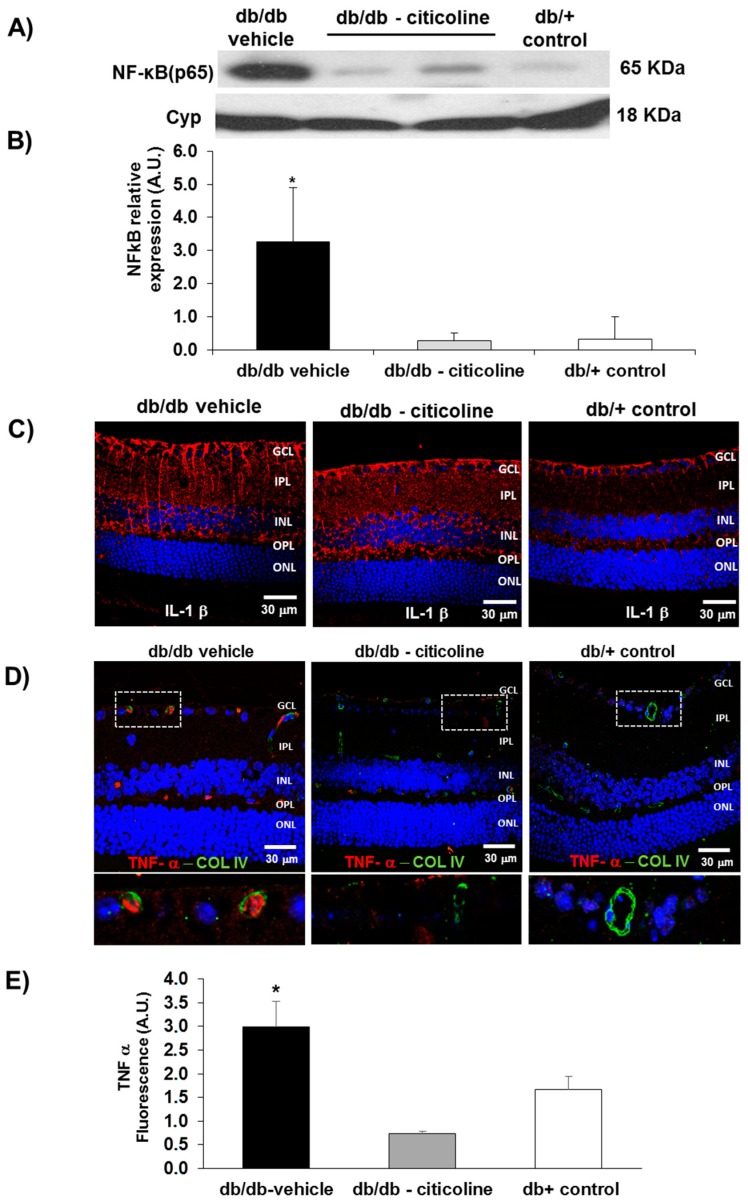
(**A**) Western blot analysis of NF-κB in a representative mouse from each group; (**B**) Quantification of western blot analysis. AU: arbitrary units. Data are expressed as mean ± SD; (**C**) IL-1β immunofluorescence (red) in a representative mouse from each group. Nuclei were labeled with Hoechst (blue); (**D**) double immunostaining for collagen IV (green), and TNF-α (red) from a representative case of a db/db mouse treated with vehicle, a db/db mouse treated with citicoline, and a non-diabetic (db/+) mouse. ONL: outer nuclear layer; OPL: outer plexiform layer; INL: inner nuclear layer; IPL: inner plexiform layer; GCL: ganglion cell layer. The dotted boxes are magnified in the bottom of panel; (**E**) quantification of TNF-α total fluorescence. *n* = seven mice per group. * *p* < 0.01 vs. the other groups.

NF-κB was significantly higher in the retina of diabetic mice in comparison with non-diabetic mice. Topical administration of liposomal formulation of citicoline resulted in a significant lowering of NF-κB (Figure 7A,B), thus reaching similar levels to found in non-diabetic control mice. 

Finally, liposomal formulation of citicoline was able to ameliorate the overexpression TNF-α, but not of IL-1β, induced by diabetes (Figure 7C–E). 

#### 2.2.3. Citicoline Had No Effect on Extracellular Glutamate Accumulation Induced by Diabetes

We find lower levels of glutamate (µmol/g proteins) in db/db mice treated with citicoline in comparison with db/db treated with vehicle, but the difference did not reach statistical significance (44.7 ± 14.2 vs. 61.1 ± 11.2; *p* = 0.22). 

## 3. Discussion

In the present study we provided evidence that topical administration of liposomal citicoline significantly prevents retinal neurodegeneration (both structural and functional) induced by diabetes in db/db mice. 

There is emerging evidence suggesting that citicoline exerts a neuropotective effect in retinal neurodegeneration induced by glaucoma [18,19,20,21]. The most diffuse hypothesis to explain the neuroprotective ability of citicoline assumes that the molecules, following injection or ingestion, are metabolized to cytidine and choline, which may be used by neuronal cells to resynthesize CDP-choline in the plasma membrane. Recently, an alternative hypothesis has been proposed, which presumes that the intact citicoline is the active agent, while choline and cytidine are less active metabolites [25]. This explanation would help clarify why citicoline is efficient in in vitro systems, where the drug is not metabolized [26]. 

In the setting of diabetic retina, Maestroni et al. [23] have recently shown that topical administration of citicoline (eye drops) exerts a neuroprotective action by preventing the narrowing of the neuroretina assessed by optical coherence tomography (OCT) in a type 1 mouse model of diabetes. However, the mechanistic pathways were not examined. Although it could be argued that eye drops can hardly reach the posterior chamber of the eye (i.e., the vitreous and the retina), this and other studies showed the contrary, and in fact, a lot of drugs are able to reach the retina in pharmacological concentrations, at least in animal models [27,28,29,30,31]. Furthermore, we used a new formulation based on liposomal citicoline, phospholipids, sodium hyaluronate, phosphate buffer, and water, in which liposomal systems would act as a penetration enhancer and to avoid the use of eye drop preservatives [24]. This new formulation might increase the patient compliance and adherence.

Since previous studies showed that citicoline led to the upregulation of synaptophysin in the penumbra region in the setting of stroke recovery [9], we wanted to investigate the effect of this molecule in the retina, a tissue embriologically brain-derived. We found that liposomal formulation of citicoline prevents the downregulation of synaptophysin induced by diabetes, thus avoiding synapsis loss. It has been previously described a reduction of synaptophysin retinal levels in streptozotocin-induced diabetic mice [32], but to the best of our knowledge there is no previous information on this issue in type 2 experimental models. It should be noted that synaptophysin is indispensable for several presynaptic functions, including the release of neurotransmitters [33,34], and therefore, the diabetes-induced reduction of synaptophysin could play an important role not only in functional abnormalities, but also in structural changes that occur in the neurodegenerative process that occurs in diabetic retina. 

In addition, we have shown that liposomal formulation of citicoline completely prevents the upregulation of NF-κB that exists in the diabetic retina. It should be noted that inflammation plays a major role in the pathogenesis of both retinal neurodegeneration and early microvascular impairment induced by diabetes [6], and NF-κB is the transcription factor which governs the production of most of proinflammatory cytokines (e.g., TNF-α, IL-6, IL-8, MCP-1) [35]. Therefore, the antiflammatory properties of citicoline are essential in its beneficial effects in early stages of DR. In addition, it is worth mentioning that citicoline may upregulate Sirtuin 1 (SIRT1) expression, which is essential for neuroprotection by means of the inhibition of NF-κB [36,37]. Notably, in the present study we provide evidence that topical administration of citicoline results in a dramatic lowering of NF-κB with no effect on IL-1β. This finding suggests that the observed decrease of NF-ĸB is a primary effect which cannot be attributable to any effect on IL-1 β. 

In close relationship with the effect on NF-κB, citicoline was able to ameliorate the perivascular overexpression of TNF-α induced by diabetes. Notably, TNF-α downregulates tight junction proteins of endothelial cells and it is also required for Vascular Endothelial Growth Factor (VEGF)-induced leakage, thus participating in the breakdown of the blood-retinal barrier (B), which is the main pathogenic factor of diabetic macular edema [38]. However, further studies to examine the effectiveness of citicoline in preventing the vascular leakage induced by diabetes are needed. 

In contrast with previous studies using various neuroprotective agents for treating DR [9,10,11,39], we did not find any effect of citicoline on the apoptosis rate in GCL and INL. At present, we do not have any plausible explanation for this observation. Moreover, the inhibition of excitotoxicity mediated by glutamate does not seem play any significant role in neuroprotection mediated by citicoline. This is an interesting finding that questions the central role of glutamate accumulation in diabetes-induced neurodegeneration, or at least indicates that neuroprotection could be achieved without any effect in the glutamate- glutamate aspartate transporter (GLAST) system. Further research to elucidate this issue seems warranted. 

In conclusion, in the present study we demonstrated that the liposomal formulation of citicoline exerts a neuroprotective effect in diabetic retina. The prevention in the downregulation synaptophysin and its anti-inflammatory properties are the main mechanisms involved. These results suggest that topical administration of citicoline in liposomal formulation could be considered as a new strategy for treating early stages of DR. 

## 4. Materials and Methods

### 4.1. Animals

The neuroprotective effect of citicoline (liposomal formulation) was tested in the db/db mouse model. This mouse carries a mutation in the leptin receptor gene and is a model for obesity-induced type 2 diabetes. We have previously characterized the neurodegenerative process in the db/db mouse model that reproduces the features of the neurodegenerative process that occurs in the human diabetic retina [40]. A total of 14 male db/db mice (BKS.Cg− + Leprdb/+ Leprdb/OlaHsd; Harlan Laboratories, Inc., Itingen, Switzerland) and 7 non-diabetic (db/+) mice matched by age (10 weeks old) were included. 

Citicoline (ophthalmic liposomal preparation, provided by OPKO (Barcelona, Spain) and Omikron (Roma, Italy), based on Citicoline 2%, phospholipids 2%, sodium hyaluronate, phosphate buffer, and water) or vehicle eye drops were administered directly onto the superior corneal surface of each eye using a syringe. In summary, one drop (5 µL) of liposomal citicoline (2%) or vehicle (5 µL of 0.9% sodium chloride) were administered twice daily for 14 days. This protocol was similarto previously reports by our group in db/db mice [11,12,13,14,15,16,17,18,19,20,21,22,23,24,25,26,27,28,29,30]. On day 15, the animals’ eyes were instilled with a drop of citicoline or vehicle approximately one hour prior to necropsy. 

On day 15, mice (12 weeks old) were euthanized by cervical dislocation and the eyes were immediately enucleated. The neuroretina from one of the eyes was extracted and frozen in liquid nitrogen and stored at –80 °C. The other eye was fixed in 4% paraformaldehyde, and routinely processed and embedded in paraffin blocks. Sections were mounted on slides and stored at 4 °C. 

This study was approved by the Animal Care and Use Committee of VHIR (Valld’Hebron Research Institute) (CEEA 75/15; September 2015). All the experiments were performed in accordance with the tenets of the European Community (86/609/CEE) and ARVO (Association for Research in Vision and Ophthalmology). 

### 4.2. Electroretinogram

Full field electroretinography (ERG) recordings were measured using the Ganzfeld ERG platform (Phoenix Research Laboratories, Pleasanton, CA, USA) as reported elsewhere [40] and following ISCEV (International Society for Clinical Electrophysiology of Vision) recommendations [41].

### 4.3. Neurodegeneration Measurements

Histological markers of neurodegeneration (glial activation and apoptosis) were evaluated by immunohistochemistry. Targets, dilution, and sources of applied primary antibodies in immunofluorescence are detailed in Table 1.

#### 4.3.1. Measurements of Glial Activation

Glial activation was evaluated by laser scanning confocal microscopy using specific antibodies against GFAP (Glial fibrillar acidic protein). Sections were fixed in acid methanol (−20 °C) for 2 min, followed by three washes with PBS, 5 min each. Sections were permeabilized with TBS-Triton X-100 0.025% and incubated in blocker (1% BSA, and 10% goat serum in PBS) for 2 h at room temperature. Sections were then incubated with rabbit anti-GFAP overnight at 4 °C in a humid atmosphere. After three washes in PBS, 5 min each, the sections were incubated with secondary antibody Alexa 488 goat-anti-rabbit (Invitrogen, San Diego, CA, USA) (1:200 dilution prepared in blocking solution). The sections were washed in PBS, counterstained with Hoestch and mounted with Mounting Medium Fluorescence (Prolong, Invitrogen) and mounted with a coverslip. Comparative digital images from samples were recorded with a Fluoview FV 1000 Laser Scanning Confocal Microscope Olympus (Olympus, Shinjuku, Tokyo, Japan) using identical brightness and contrast settings. 

To evaluate the degree of glial activation a scoring system based on the extent of GFAP staining previously described was used [42]. This scoring system is as follows: Müller cell endfeet region/GCL only (score 1); Müller cell endfeet region/GCL plus a few proximal processes (score 2); Müller cell endfeet plus many processes, but not extending to ONL (score 3); Müller cell endfeet plus processes throughout with some in the ONL (score 4); Müller cell endfeet plus lots of dark processes from GCL to outer margin of ONL (score 5).

#### 4.3.2. Apoptosis Assessment

The TUNEL (Terminal Transferase dUTP Nick-End Labeling) staining was carried out using the DeadEnd Fluorometric TUNEL System kit (PROMEGA, Madison, WI, USA). Cryosections of retina were permeabilized by incubation for 2 min on ice with 0.1% Triton X-100 in 0.1% sodium citrate, freshly prepared. The secondary antibody was Alexa 488 goat-anti-rabbit (Invitrogen, San Diego, CA, USA). For evaluation by laser scanning confocal microscopy the excitation wavelength was 488 nm (detection in the range 515–565 nm (green)). 

Caspase-3 was evaluated by immunofluorescence. Paraffined sections were deparaffinized in xylene and rehydrated in ethanol. Sections were fixed in acid methanol (−20 °C) for 1 min and washed with 0.01 M 4 phosphate buffered saline (PBS) at pH 7.4. Then, sections were incubated in blocking solution (10% NGS, 0.1% Triton X-100, PBS) for 1 h at room temperature and afterwards, they were incubated with a specific primary antibody overnight at 4 °C. The following day, after washing, sections were incubated with a fluorescent ALEXA 594 as a secondary antibody (anti-rabbit) (Life Technologies S.A, Madrid, Spain) in blocking solution for 1 h and washed. Finally, nuclei were stained with Hoechst and mounted in Mounting Medium Fluorescence (Prolong, Invitrogen) with a coverslip. Images were acquired with a confocal laser scanning microscope (FV1000; Olympus, Hamburg, Germany). Five fields (three corresponding to the central and two to the peripheral retina) from each section were analyzed. The same locations and number of fields were measured in all retinas. Fluorescence intensity of images was quantified by ImageJ.

### 4.4. Methods Used in Order to Explore the Mechanisms of Action

#### 4.4.1. Glutamate Quantification

Quantification of glutamate was performed by reverse phase ultra-performance liquid chromatography (UPLC) (Acquity-UPLC, Waters, Waters, Milford, MA, USA) as aminoquinoline derivatives (AccQ-Tag chemistry, MassTrak AAA method and instruments, Waters, Milford, MA, USA), following the methodology previously described by Narayan et al. [43].

#### 4.4.2. Assessment of Synaptophysin

The same methodology above described for caspase-3 analysis was used. The primary antibody used was a rabbit monoclonal anti-synaptophysin antibody. 

#### 4.4.3. Assessment of NF-κB

Proteins from the neuroretina were extracted in 80 μL of lysis buffer (RIPA buffer) and 1× protease inhibitor cocktail (Sigma, St. Louis, MO, USA). A total of 25 μg protein was resolved by 10% SDS-PAGE and transferred to a PVDF membrane (Bio-Rad Laboratories, Hercules, CA, USA). The primary antibody mouse monoclonal anti-NF-κB (p. 65) (1:1000; sc-8008; Santa Cruz, Dallas, TX, USA) was incubated overnight at 4 °C, and on the following day, the secondary antibody anti-mouse HRP (1:10,000; P0260; Agilent Dako, Santa Clara, CA, USA) was incubated for 1 h at room temperature. Anti-cyclophilin A (1:10,000; BML-SA296; Enzo, Farmingdale, NY, USA) was used to normalize protein levels. Densitometric analysis of the autoradiographs was performed with ImageJ software.

#### 4.4.4. Proinflammatory Cytokines

Interleukin 1β (IL-1β) and Tumor Necrosis Factor α (TNF-α) were assessed by immunofluorescence (antibodies and dilution are shown in Table 1). 

### 4.5. Blood-Retinal Barrier (BRB) Function 

Retinal thickness was assessed by Spectral Domain OCT (Image-Guided Tomographer integrated to Micron III. Phoenix Research Labs, Pleasanton, CA, USA). The sealing function of the blood retinal barrier was analyzed by determining the leakage of albumin by immunohistochemistry (Olympus FluoView™ FV1000 Confocal Microscope, Hamburg, Germany).

### 4.6. Statistical Analysis

Statistical comparisons were performed with Student’s unpaired *t* tests. For multiple comparisons, one-way ANOVA followed by the Bonferroni test was used. The Fisher’s exact test was used to analyze categorical variables. Levels of statistical significance will be set at *p* < 0.05.

## Figures and Tables

**Table 1:** Targets, dilution, and sources of applied primary antibodies (Abcam, Cambridge, UK) in immunofluorescence.

Target Molecule	Clone	Dilution	Manufacturer
GFAP	Rabbit polyclonal	1/500	Abcam (ab7260)
Cleaved caspase 3	Rabbit polyclonal	1/200	Abcam (ab3623)
Synaptophysin	Rabbit monoclonal	1/100	Abcam (ab32127)
GLAST	Rabbit polyclonal	1/100	Abcam (ab416)
IL-1β	Rabbit polyclonal	1/100	Abcam (ab9722)
TNF-α	Mouse monoclonal	1/100	Abcam (ab8348)
Serum Albumin	Sheep polyclonal	1/500	Abcam (ab8940)
